# Impact of intensive training on mental health, the experience of Port Said, Egypt

**DOI:** 10.1186/s13033-021-00461-3

**Published:** 2021-04-15

**Authors:** Saverio Bellizzi, Amal Khalil, Ahmed Sawahel, Alessandra Nivoli, Liliana Lorettu, Dina Sabry Said, Susanna Padrini

**Affiliations:** 1 Independent Consultant, Geneva, Switzerland; 2 Nursing Faculty, Port Said, Egypt; 3 Helwan Mental Health Hospital, Cairo, Egypt; 4 University of Sassari, Sassari, Italy; 5 College of Business Administration, American University of the Middle East, Eqaila, Kuwait; 6 A.I.S.P.O. Associazione Italiana per la Solidarietà tra i Popoli, Milan, Italy

## Abstract

**Background:**

Mental disorder is extremely common globally and integration of mental health in primary health services represents a critical gap especially in low- and middle-income Countries like Egypt. The World Health Organization has repeatedly called for effective training and support of primary care providers in the identification and treatment of mental health problems over the last decades.

**Methods:**

This paper aimed to evaluate attitudes and knowledge of health care providers toward mentally ill patients and measure knowledge and retention of training messages over time. A 3-day mental health training workshop for nurses of public health facilities in the Governorate of Port Said was organized. Pre-training and post-training questionnaires (immediately after the workshop and 3 months later) were used. Significance of gain in scores was examined between baseline and following cross sectional rounds.

**Results:**

The 73 participants in the study revealed a statistically significant improvement in knowledge and attitude toward mental health from the baseline (pre-training), from a general mean score for desirable answers of 10.5 (± 1.2) to 21.2 (± 0.6). However, results slightly declined three months after from the workshop (18.5 (± 0.6)).

**Conclusions:**

Intensive short-term training on mental illness could be instrumental in improving knowledge and attitudes in countries like Egypt with extensive needs in terms of quality of comprehensive healthcare at primary and secondary level. However, additional evidence is needed to improve retention of information over time and to translate knowledge into clinical practice.

## Introduction

Mental disorder is extremely common in all countries, both in high-resourced and in low-resourced settings. While 14% of the global burden of disease is attributed to these disorders, most of the people affected, 75% in many low-income countries, do not have access to the treatment they need [1].

Impact of long-standing disability due to mental health conditions is huge and integration of mental health in primary health services represents a critical gap in several countries, especially low- and middle-income countries (LMICs) [2, 3].

Mental health services in Egypt are characterized by a substantial emphasis on hospitals with insufficient attention given to integrating mental health into primary care [4]. This leads to inadequate prevention, early detection as well as poor rehabilitation and social inclusion. One of the critical consequences is also represented by the lack of expertise on mental health issues across practitioners including family physicians, nurses and social workers [4].

The World Health Organization (WHO) has repeatedly called for effective training and support of primary care providers in the identification and treatment of mental health problems over the last several decades [5, 6].

Mental health services face a number of challenges in Egypt, and a number of projects have sought to integrate mental health in the public health system [4]. The scale of training in medical schools and other training institutions does not reflect the importance of this field as contributor to morbidity. On the other hand, most resources are allocated to a few large centralized psychiatric hospitals, with inadequate availability of beds for acute inpatient care provision, especially due to the fact that around 60% of beds are occupied by long stay patients [7].

We organised a 3-day mental health training workshop for nurses of several primary and secondary level public health facilities, including the Psychiatric Hospital of Port Said, in the Governorate of Port Said, Egypt, in order to train them in core skills such as communication, assessment, mental state examination, diagnosis, and management of patients with mental illnesses. We also distributed questionnaires to the study-participants to pursue a three-fold objective of: (1) evaluating (pre-training) attitudes and knowledge of health care providers toward mentally ill patients; (2) to measure knowledge after the training; and (3) to explore the retention of training messages over time.

The main objective of this analysis was twofold. Specifically, we aimed to evaluate the baseline attitudes and knowledge of non-psychiatric nurses against those of psychiatric nurses at a different health facility as well as the retention of knowledge over time across the two categories of nurses.

## Methods

### Design

This is an observational analytical follow-up study. We organised a 3-day mental health training workshop for nurses of Public Health facilities in the Governorate of Port Said, in order to train them in core skills such as communication, assessment, mental state examination, diagnosis, and management of patients with mental illnesses.

In order to assess the retention of information provided during the training, the same questionnaire is distributed and administered (1) before the training, (2) immediately and (3) three months after the training.

### Sample

This study was carried out amongst nurses, operating in public health facilities of the Port Said Governorate, who already participated in previous courses organized by the Italian Cooperation. While sample size calculation was not performed, we invited all the available nurses from the Port Said public health services in accordance with availability and working time. While all the 73 participants took the training, pre-test and first post-test in April 2018, only 49 (67.1 %) took the second test post-training in September 2018 (Table 1).

**Table 1:** Distribution of nurses by health facility

Health facility	N (%)		
	Pre-test	Post-test	Post-test after 3 months
Primary Health Centers	14 (19.2)	14 (19.2)	9 (18.4)
Port Said Dental Hospital	5 (6.8)	5 (6.8)	2 (4.1)
El Ahrab Hospital	2 (3.1)	2 (3.1)	2 (4.1)
El Masah El Bahary Hospital	3 (4.1)	3 (4.1)	3 (6.2)
El Nasr Hospital	4 (5.1)	4 (5.1)	3 (6.2)
Port Said Epidemic Hospital	3 (4.1)	3 (4.1)	3 (6.2)
Port Said Geriatric Medical Centre	3 (4.1)	3 (4.1)	3 (6.2)
Port Said Ophthalmology Hospital	7 (9.6)	7 (9.6)	2 (4.1)
Port Fouad General Hospital	9 (12.3)	9 (12.3)	5 (10.2)
Port Said General Hospital	6 (8.2)	6 (8.2)	6 (12.1)
Port Said Psychiatric Hospital	11 (15.1)	11 (15.1)	7 (14.0)
Zohour Hospital	5 (6.8)	5 (6.8)	3 (6.2)
Port Said Obstetric Hospital	1 (1.5)	1 (1.5)	1 (2.0)

### Training

The training package was adapted using the modules for the training of primary care workers in low- and middle-income countries utilized in countries like Kenya and Iraq [8, 9]. The package is highly structured into five overall units. The first focuses on core concepts (mental health and mental disorders, and their contribution to physical health, economic and social outcomes). The second addresses core skills such as communication, assessment, mental state examination, diagnosis, management, managing difficult cases, management of violence and breaking bad news. The third covers common neurological disorders such as epilepsy and dementia while the fourth focuses on psychiatric disorders following the World Health Organization primary care guidelines. Finally, the fifth deals with policy and legislation around integration of mental health into annual operational plans. Other common severe disorders were discussed, including schizophrenia and bipolar disorder [10].

Each unit was subdivided into a series of 30-min modules delivered by trained Psychiatrists. The course was administered over 3 days and consisted of a combination of lectures, case studies and problem-solving scenarios, and took place in the month of April 2018.

### Questionnaire

Each participant was invited to fill a questionnaire, based on previous studies [2, 11], including several aspects of the syllabus of the training (Table 2). Specifically, the questionnaire consists of 25 statements and for each item the respondent is required to state whether he agrees or disagrees. The questionnaire was pre-tested and validated among a small sample of nurses of the Port Said Nursing Institute.

**Table 2:** Questionnaire

Item	Desirable answer	Code answer
1. Health is absence of illnesses	Disagree	1
2. Depression is a form of disability	Agree	1
3. Criteria of Schizophrenia include double or multi-personality	Disagree	1
4. Psychiatric patients are usually aggressive and represent a danger for himself and for others	Disagree	1
5. Psychiatric medication cause addiction	Disagree	1
6. Delusion is the hallucination of schizophrenia patients	Disagree	1
7. Headache, stomachache, fatigue, muscle pain are symptoms of depression	Agree	1
8. Psychiatric nurses are always subject to verbal or physical aggression during care for psychiatric patient	Disagree	1
9. To improve depressed patient status, advice to be more religious, to pray, and to appreciate positive sides of life	Disagree	1
10. Stop working, lack of concentration, and insomnia, and being sarcastic towards other people, and non-flexibility on others’ opinion, are some of the criteria for maniac episodes	Agree	1
11. Electro-convulsive therapy (ECT) is not a safe treatment for mental ill patients	Disagree	1
12. Before diagnosing a psychiatric patient we need to exclude the following: HIV, Hypothyroidism, DM, cerebral palsy	Agree	1
13. No need to measure vital sign for psychiatric patient unless he is diagnoses with a physical disease	Disagree	1
14. Reading Quran and prayer will cure psychiatric illness	Disagree	1
15. Nurses could support and help depressive patient by reporting stories of other patients in worse conditions	Disagree	1
16. Psychiatric nurse assessment includes physical appearance, social status, stuttering, and lab investigation	Agree	1
17. Psychiatric hospital is the best place to treat psychiatric patients	Disagree	1
18. Psychiatric patient needs special care that is not available in general hospital	Disagree	1
19. Neglect answering question is the best way to deal with anxious patient	Disagree	1
20. Most of psychiatric diseases are because of lack of faith and not being religious	Disagree	1
21. Religious clergy are the best ones to treat obsessive compulsive patients (OCD)	Disagree	1
22. Schizophrenia percentage increases in lower income society more than high income society	Disagree	1
23. We should not ask depressed patient about suicidal thoughts so he will not commit it	Disagree	1
24. Family and social support are very important to support psychiatric patients	Agree	1
25. Psychiatric diseases are considered chronic diseases like DM and hypertension	Agree	1

The workshop was conducted in the Port Said Nurses Institute and was supported by the Egypt Ministry of Health and by the Italian Cooperation.

### Data collection and statistical analysis

Data were entered by a medical epidemiologist in Excel files, which were uploaded for analysis using the software Stata/MP v.14.

Desirable responses for each item in the questionnaire (Table 1) was given a score of 1 while undesirable responses will be given a score of 0. Score before and after training, as well as after 3 months was calculated. Significance of gain in scores was examined between baseline and following cross sectional rounds using Paired T-Student test.

## Results

### Pre‐training test

The general mean score for desirable answers per participant was 10.5 (± 1.2) and ranged from a minimum of 3 up to a maximum value of 18 (consider that the maximum score would have been 25). The mean score for desirable answers in NON psychiatric nurses was 9.4 (± 1.4) and ranged from a minimum of 3 up to a maximum value of 18. The mean score for desirable answers in psychiatric nurses was 13.9 (± 1.0) and ranged from a minimum of 9 up to a maximum value of 18. The most problematic items were the no. 3 (criteria of Schizophrenia include double or multi-personality), the no. 6 (delusion is the hallucination of schizophrenia patients), the no. 8 (psychiatric nurses are always subject to verbal or physical aggression during care for psychiatric patient), and the no. 18 (psychiatric patient need special care that is not available in an general hospital), which respectively totalled 4, 6, 3, and 6 correct answers. The least problematic items were the no. 7 (headache, stomach ache, fatigue, muscle pain are symptoms of depression), and the no. 24 (family and social support are very important to support psychiatric patients), which totalled 39 and 43 corrects answers.

### Post‐training tests

As shown in Table 3, knowledge around mental health significantly increased when comparing the overall score for each of the 25 items included in the questionnaire before and immediately after the 3-day training. Item no. 19 (“*Neglect answering question is the best way to deal with anxious patient*”) is the only subject that did not register a statistically significant increase in knowledge (p = 0.06).

**Table 3:** Differences in participants’ attitudes to mental health issues before and after training

Total desirable answers achieved	Pre-test (73 participants)	Post-test (73 participants)	P-value (Pre vs. Post)	Post-test after three months (49 participants)	P-value (Post vs. 3 months)
Item 1	24	68	< 0.05	39	0.04
Item 2	25	51	< 0.05	21	< 0.05
Item 3	12	58	< 0.05	30	0.03
Item 4	26	50	< 0.05	32	0.7
Item 5	32	63	< 0.05	41	0.8
Item 6	22	61	< 0.05	30	< 0.05
Item 7	62	70	0.04	34	< 0.05
Item 8	10	32	< 0.05	19	0.07
Item 9	19	64	< 0.05	41	0.8
Item 10	50	69	0.03	44	0.09
Item 11	34	72	< 0.05	43	0.06
Item 12	41	50	0.04	26	0.03
Item 13	57	71	0.02	43	0.04
Item 14	20	67	< 0.05	45	0.9
Item 15	27	68	< 0.05	36	0.03
Item 16	52	67	0.03	41	0.05
Item 17	21	63	< 0.05	32	< 0.05
Item 18	12	36	< 0.05	28	0.07
Item 19	50	63	0.06	45	0.4
Item 20	33	70	< 0.05	44	0.9
Item 21	48	70	< 0.05	46	0.8
Item 22	49	66	0.05	40	0.1
Item 23	18	58	< 0.05	29	< 0.05
Item 24	67	68	0.4	43	0.3
Item 25	32	70	< 0.05	31	< 0.05

On the other hand, the questionnaire administered after three months showed a significant decline in knowledge for six items: *“depression is a form of disability”, “delusion is the hallucination of schizophrenia patients”, “headache, stomach ache, fatigue, muscle pain are symptoms of depression”, “psychiatric hospital is the best place to treat psychiatric patients”, “we should not ask depressed patient about suicidal thoughts so he will not commit it”, and “psychiatric diseases are considered chronic diseases like DM and hypertension”* (Table 3).

The first post-training test general mean score for desirable answers per participant was 21.2 (± 0.6) and ranged from a minimum of 14 up to a maximum value of 24. The mean score for desirable answers in NON psychiatric nurses was 20.0 (± 0.7) and ranged from a minimum of 14 up to a maximum value of 24. The mean score for desirable answers in psychiatric nurses was 21.8 (± 0.3) and ranged from a minimum of 16 up to a maximum value of 22. More than 20 participants replied correctly for all items except for no. 8 (psychiatric nurses are always subject to verbal or physical aggression during care for psychiatric patient) for which only 10 nurses answered correctly, and the no. 18 (psychiatric patient need special care that is not available in general hospital) for which only 16 did. Almost all participants correctly answered to item no. 1 (health is absence of illnesses), no. 7 (headache, stomach ache, fatigue, muscle pain are symptoms of depression), no. 10 (stop working, lack of concentration, and insomnia, and being sarcastic towards other people, and non-flexibility on others’ opinion, are some of the criteria for maniac), no. 11 [electro-convulsive therapy (ECT) is not a safe treatment for mental ill patients], no. 13 (no need to measure vital sign for psychiatric patient unless he is diagnoses with a physical disease), no. 20 (most of psychiatric diseases are because of lack of faith and not being religious), and no. 25 (psychiatric diseases are considered chronic diseases like DM and hypertension).

The second post-training test general mean score for desirable answers was 18.5 (± 0.7) and a similar decline was noted for both groups, psychiatric nurses and NON psychiatric nurses, which recorded respectively 18.9 (± 0.6) and 18.1 (± 0.6).

## Discussion

This study showed the potential value of training nurses of different departments and operating at both primary and secondary health level. The statistically significant improvement in acquisition of knowledge and positive attitudes towards mental disorders is a fundamental finding of this study. Such a combination (knowledge and attitudes) is indeed is critical for quality and efficient service delivery.

Similar results were indicated by previous reports that examined changes in knowledge and attitude of primary healthcare workers and primary care physicians after short-term trainings [10, 12–14].

A WHO collaborative study also showed an equal magnitude increase in knowledge and behavior of general health workers in six different low- and middle-income countries despite approaches to training varied between study areas [15]. The same study emphasized the persistence of knowledge and attitude for 18 months post training, which is in line with our study in spite of a slight decline.

As correctly inferred by Ignacio et al., some culturally ingrained beliefs are reflected in specific negative attitudes and may require longer term interventions to reverse them [15]. In the case of our experience in Port Said, this is particularly true for the items “psychiatric nurses are always subject to verbal or physical aggression during care for psychiatric patient”, and “psychiatric patient need special care that is not available in general hospital”, for both of which the correct answer is No.

While a general improvement in knowledge was registered, is also important to remark how no significant changes were detected for some items, which remained similar to the baseline scores. This was showed also by Chinnayya et al. [11] and reinforces the theory of culturally ingrained beliefs and attitudes, which might require alternative and longer strategies of training.

It is essential to embed mental health knowledge and skills within primary and secondary care and the integration of mental health into the basic training of staff would be fundamental in association with post basic training and continuing professional development and for building independent mental health researchers [16].

As very well-emphasized by Makanjuola et al. [2] there is “no health without mental health” and overall service delivery would drastically benefit from the inclusion of mental health knowledge and positive attitudes. In fact, essential universal health would be an unattainable goal if the complex relationship between physical and mental health is not addressed at the healthcare service delivery portal level [17–19].

While the protocol for our study was substantiated by a literature review and each step was carefully implemented, a few limitations are present.

First of all, this study did not explore whether the skills acquired would impact on clinical practice. However, some studies have reported significant improved skill changes in workplaces after intensive training with similar approaches [13]. Secondly, although questionnaires were anonymous and completely confidential, changes were obtained by self-report and may have been influenced by response bias rather than reflecting a “true conviction”. Finally, despite a telephone attempt to track those who did not complete the second post-training, none undertook the questionnaire on another possible date, thus representing a potential source of knowledge bias.

To conclude, intensive short-term training on mental illness could be instrumental in improving knowledge and attitudes in countries like Egypt with extensive needs in terms of quality of comprehensive healthcare at primary and secondary level. However, retention of information seems to decline overtime. Further studies are warranted that are tailored to local contexts in order to investigate how such programs translate into clinical practice.

## Data Availability

Data are available under request.
